# Interaction between voice-gender difference and spatial separation in release from masking in multi-talker listening environments

**DOI:** 10.1121/10.0005831

**Published:** 2021-08-05

**Authors:** Yonghee Oh, Sarah E. Bridges, Hannah Schoenfeld, Allison O. Layne, David Eddins

**Affiliations:** 1 Department of Speech, Language, and Hearing Sciences, University of Florida, Gainesville, Florida 32610, USA; 2 Department of Communication Science and Disorders, University of South Florida, Tampa, Florida 33612, USA yoh@phhp.ufl.edu, sbridges1@ufl.edu, hschoenfeld@ufl.edu, allisonlayne@ufl.edu, deddins@usf.edu

## Abstract

Voice-gender difference and spatial separation between talkers are important cues for speech segregation in multi-talker listening environments. The goal of this study was to investigate the interactions of these two cues to explore how they influence masking release in normal hearing listeners. Speech recognition thresholds in competing speech were measured, and masking release benefits by either voice-gender difference or spatial separation cues were calculated. Results revealed that the masking releases by those two cues are inversely related as a function of spatial separation, with a gender-specific difference of transition between the two types of masking release.

## 1. Introduction

Speech perception in noisy listening environments is challenging for all listeners and even more so for persons with auditory deficits associated with age, hearing loss, or their combination. Listening environments comprised of multiple simultaneous talkers are of particular interest because of the additional challenges associated with separating and then selectively focusing on the talker currently of interest to the listener. This multi-talker listening situation is often referred to as the “cocktail party” phenomenon (Cherry, 1953). It is well established that segregating and attending to speech in this type of background noise is exceptionally difficult and that human listeners with normal cognitive and perceptual abilities are remarkably good at cocktail party listening. One of the greatest sources of difficulty is the abundance of unique talker characteristics that are in competition with a desired speech message (target). These lead to energetic and informational masking that interferes with the encoding and understanding of the target. If that masking becomes distracting enough, the listener's ability to identify the target can be substantially reduced. For this reason, it is important to understand in detail the acoustic cues used in the cocktail party listening environment to gain a more comprehensive understanding of both the processes and the techniques that listeners rely on to understand a specific speech target for the task of communication.

In multi-talker listening situations, also known as “speech-on-speech masking,” two major acoustic cues that can enhance the speech segregation performance of normal hearing (NH) listeners are (1) differences in voice characteristics among talkers (e.g., characteristic fundamental frequency differences between male and female talkers) and (2) spatial separation between target and competing talkers (e.g., co-located talkers versus spatially separated target and competing talkers; Allen *et al.*, 2008; Brungart, 2001; Brungart *et al.*, 2001; Best *et al.*, 2012; Darwin *et al.*, 2003; Ericson *et al.*, 2004; Gallun *et al.*, 2013; Gaudrain and Başkent, 2018; Humes *et al.*, 2017; Mackersie *et al.*, 2011; Srinivasan *et al.*, 2016). This enhancement is referred to as release from masking. In the current study, we systematically investigate potential interactions between these two distinct cues to explore how they influence the relative magnitude of masking release. In natural communication scenarios, there are a variety of other cues used for segregating speech-on-speech masking that are not considered here, from low-level acoustic features like co-modulation to higher-level linguistic cues such as word familiarity and neighborhood density.

### 1.1 Voice gender release from masking

Two unique vocal characteristics contribute most to the distinct perception of individual talkers: vocal pitch height (ranging from low to high and associated with fundamental frequency) and vocal timbre (an attribute of voice quality). Pitch height is the perceptual correlate of the fundamental frequency (F0) of the voice and is proportional to the rate and periodicity of vocal fold vibrations. Controlled variations in F0 lead to changes in perceived pitch height that can convey important prosodic information that gives rise to semantic context and include the concepts of intonation or stress. Another voice characteristic, timbre, is determined by vocal tract length (VTL). Timbre is an interesting component of voice, as it cannot be defined or measured by a single physical dimension. Rather, it can be thought of as the auditory element that distinguishes two voices as being dissimilar even when they are equal in pitch height, loudness, and duration.

Differences in these unique voice characteristics are most readily found among talkers of different genders but also exist between talkers of the same gender. Differences in voice characteristics between talkers of different genders lead to greater masking release than differences in voice characteristics between talkers of the same gender (e.g., Brungart, 2001; Brungart *et al.*, 2001; Ericson *et al.*, 2004). Oh and Reiss (2017) referred to this benefit as “voice gender release from masking” (VGRM), where “gender” denotes the classical categorization of a talker's voice with their assigned sex at birth. In those investigations, when the target and masker talkers were different genders, percent correct target identification was 15%–20% points higher than conditions with same-gender talker and maskers. Other studies have investigated the influence of F0 and VTL, two of the primary physical differences present between voices of different genders. Talker differences created by parametric manipulation of F0 or VTL alone in synthetic speech also produce masking release; however, performance increases associated with these singular manipulations are less than the performance increases observed with the full complement of acoustic cues of natural voices. This indicates that in natural voices, other vocal characteristics such as voice quality (i.e., breathiness, roughness, and strain) and intonation pattern can also contribute significantly to the overall VGRM (Allen *et al.*, 2008; Darwin *et al.*, 2003; Gaudrain and Başkent, 2018; Mackersie *et al.*, 2011).

### 1.2 Spatial release from masking

Increasing differences in the apparent source location of the target and competing speech facilitates segregation of concurrent speech. This facilitation is referred to as “spatial release from masking” (SRM). The most common SRM paradigm presents the target and competing speech from a single co-located reference condition or the target speech from one source location (i.e., 0° azimuth) and the competing speech from one or more spatially separated source locations (i.e., asymmetrical or symmetrical spatial configuration) in comparison conditions. Spatial separation of the target and masker speech can dramatically enhance the ability to understand the target speech relative to the co-located condition.

Listeners with NH can achieve significant SRM in a variety of different spatial configurations. Generally, greater spatial separation between target and competing speech produces greater SRM, but performance can be affected by various factors. Subject factors such as age, hearing loss, or their combination are associated with significant reductions in SRM (Best *et al.*, 2012; Gallun and Diedesch, 2013; Humes *et al.*, 2017). SRM also declines with increasing numbers of competing talkers (Brungart *et al.*, 2001; Humes *et al.*, 2017; Yost, 2017). In addition, target speech identification depends upon target and masker similarity, including uncertainty in the spectral and temporal domains (Brungart *et al.*, 2001; Eddins and Liu, 2012; Hawley *et al.*, 2004; Kidd *et al.*, 2010; Litovsky, 2012). Less spectral or time overlap between target and masker speech can yield greater SRM (i.e., energetic masking). Confusion of masker speech content with target speech content can reduce SRM (i.e., informational masking).

Previous investigations of SRM using speech stimuli have included a variety of talker and masker parameters and characteristics. However, the interaction between SRM and talker voice gender has not yet been investigated in detail. Gallun and Diedesch (2013) investigated spatial release from masking with talkers of different genders and showed (their Fig. 1) thresholds that decrease in target-to-masker ratio (TMR) across spatial separations to a greater extent for the same-gender conditions (∼7 dB) than for the different-gender conditions (∼2 dB). Gallun and Diedesch (2013) focused only on SRM and variations in SRM with respect to talker gender differences and did not consider VGRM. Importantly, their analyses did not consider potential interactions between the individual contributions of spatial cues and voice-gender cues. Knowledge of these interactions is essential to fully account for the release from masking one should expect with any given combination of spatial separation and voice-gender difference.

**Fig. 1.**
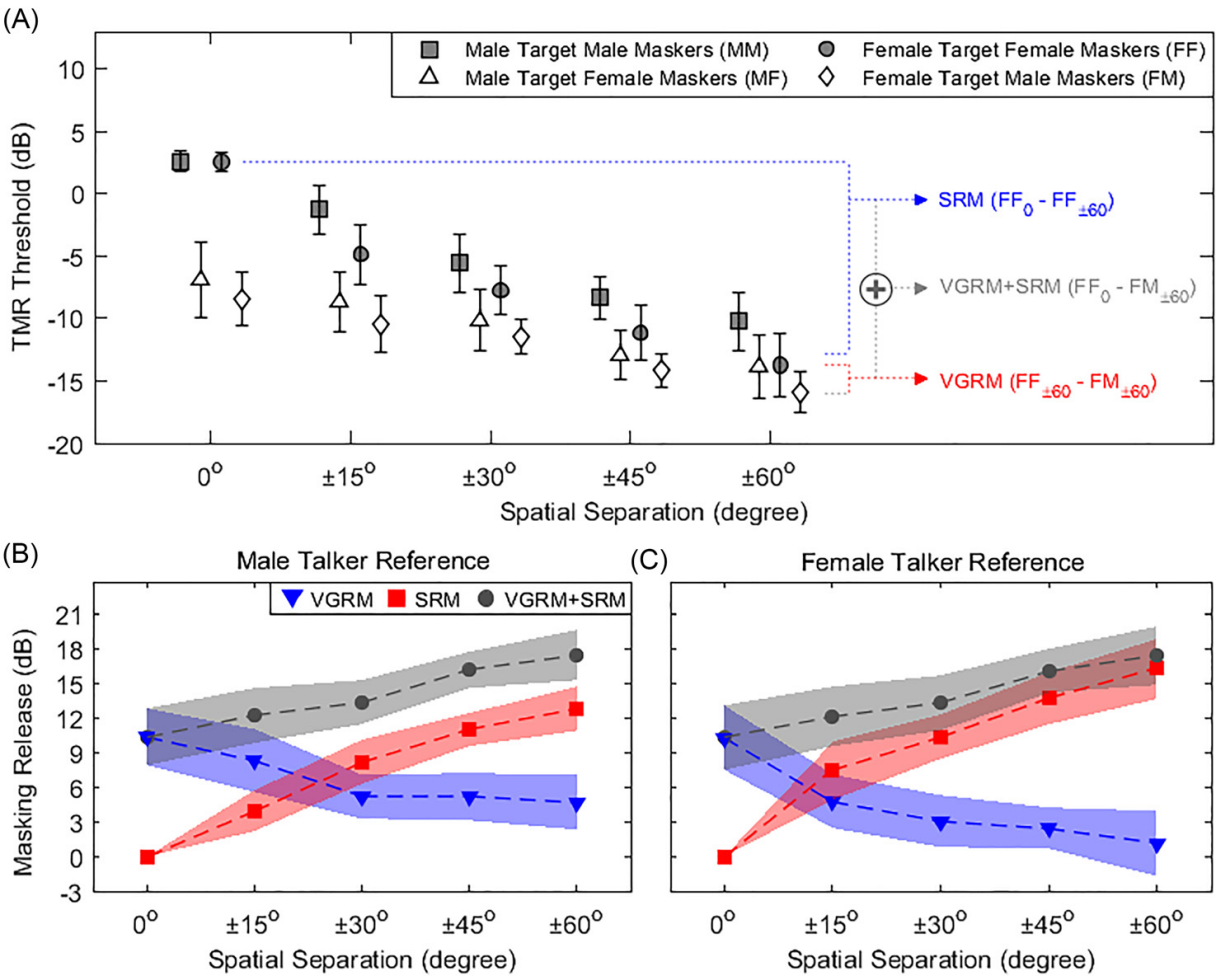
Speech recognition thresholds and masking releases due to VGRM and SRM between target and maskers in the speaker array setup. (A) Average TMRs as a function of target-masker spatial separation (0°, ±15°, ±30°, ±45°, ±60°) for four different gender differences between target and maskers (MM, MF, FF, and FM). (B) Average masking releases in male target-masker conditions: VGRM (MM_k_ − MF_k_ or MM_k_ − FM_k_: blue triangles), SRM (MM_0_ − MM_k_: red squares), and VGRM + SRM (MM_0_ − MF_k_ or MM_0_ − FM_k_: gray circles). (C) Average masking releases in female target-masker conditions: VGRM (FF_k_ − FM_k_ or FF_k_ − MF_k_: blue triangles), SRM (FF_0_ − FF_k_: red squares), and VGRM + SRM (FF_0_ − FM_k_ or FF_0_ − MF_k_: gray circles). “k” indicates target-masker spatial separation (0°, ±15°, ±30°, ±45°, ±60°). Error bars and shaded areas represent standard deviation of the mean.

The primary purpose of the present study was to establish the nature of the combined effects of both VGRM and SRM on the total masking release by systematically varying spatial separation of target and masker talkers and voice-gender differences between competing talkers. Testing of young listeners with NH will establish how the “typical” auditory system makes use of both monaural (VGRM) and binaural (SRM) cues separately and simultaneously to achieve masking release. We hypothesized a trading relationship between the perceptual weight applied to voice-gender difference and spatial separation cues. We expected that masking release would be maximized when both VGRM and SRM cues were present between target and masker talkers. Further, listeners would rely more heavily on voice characteristic differences when the contributions to masking release from spatial separation are minimal, and the weight between the two cues would shift to spatial separation as spatial separation extent increased.

## 2. Methods

### 2.1 Subjects

Twenty young adult listeners (18 females; mean age = 22.4 ± 1.8 years) participated in this study. NH listeners had audiometric thresholds <25 dB hearing level (HL) at octave frequencies between 125 and 8000 Hz. Average thresholds across frequency were 5.1 ± 6.4 dB HL for the left ear and 5.9 ± 6.9 dB HL for the right ear. All subjects scored ≥27 on the Mini-Mental State Examination (MMSE; Folstein *et al.*, 1975), ruling out cognitive impairment that would potentially influence performance. All experiments were conducted according to the guidelines for the protection of human subjects as set forth by the institutional review board (IRB) of the University of Florida, and the methods employed were approved by that IRB.

### 2.2 Stimulus materials

Target and masker speech were drawn from the coordinate response measure (CRM; Bolia *et al.*, 2000) speech corpus, which is one of the most popular English speech corpora for measuring SRM. Each sentence of the CRM speech corpus consists of the same syntactic structure in the form “Ready [*call sign*] go to [*color*] [*number*] now.” There are eight *call signs* (“Arrow,” “Baron,” “Charlie,” “Eagle,” “Hopper,” “Laker,” “Ringo,” “Tiger”), four *colors* (“blue,” “green,” “red,” “white”), and eight *numbers* (1–8). The corpus includes all possible *call sign*, *color*, and *number* combinations spoken by four male and four female talkers, leading to 2048 unique speech samples (256 CRM phrases for each talker).

### 2.3 Procedure

The experiment was conducted in a single-walled, sound-attenuating booth. Speech stimuli, stored as .wav files with a sampling rate of 44.1 kHz, were presented using custom matlab (version R2018b, MathWorks, Natick, MA) scripts. Stimuli were routed through an RME UFX+ audio interface (RME Audio, Haimhausen, Germany) and delivered via frequency-equalized Yamaha HS5 loudspeakers (Yamaha, Shizuoka, Japan). A total of nine loudspeakers were separated by 15° in the horizontal plane and positioned in the front hemifield a distance of 1.5 m from the center of the listener's head. The output of all loudspeakers was calibrated using a Brüel & Kjær sound level meter with an A-weighting filter (Brüel & Kjær Sound & Vibration Measurement A/S, Nærum, Denmark).

Each listener was presented with three simultaneous phrases from the CRM corpus (one target phrase and two simultaneous masker phrases). The task was to report the key words (*color* and *number*) in the target phrase, which was indicated by the call sign “Charlie.” The target phrase was fixed at 0° azimuth. In different conditions, presentation of the two masker phrases was either collocated with the target phrase (0°) or from left and right loudspeakers at progressively greater spatial separations (±15°, ±30°, ±45°, and ±60°). Symmetric target-masker separation minimized the availability of any better ear cue due to the head shadow effect (Shaw, 1974; Marrone *et al.*, 2008) and thus maximized the potential to use spatial cues or voice cues for source segregation. Both masker phrases presented contained randomized combinations of the *call sign*, *color*, and *number*. To prevent confusion, no maskers contained the call sign “Charlie,” and none of phrases had common color or number key words on a single trial.

In addition to the five spatial separations, four different voice gender target-masker combinations were tested: MM (male target, male maskers), MF (male target, female maskers), FF (female target, female maskers), and FM (female target, male makers). To improve reliability, each condition was tested twice, for a total of 5 spatial separations × 4 gender differences × 2 repetitions = 40 separate measures. All gender differences and spatial configurations were tested in a random order to reduce any listener predictability.

Listeners were instructed to face the front speaker and attend to the target sentence, always identified by the call sign “Charlie.” After each stimulus presentation, listeners selected target color and number keywords from a grid of 32 possible color/number combinations displayed on an iPad tablet computer (Apple, Cupertino, CA). Immediate feedback was provided at the top of the grid-array with text reading “correct” or “incorrect.”

The target from the CRM corpus was presented at a fixed comfortable listening level of 60 dBA to ensure listener audibility. The presentation level of the masker sound was adjusted after each trial using a one-up, one-down, adaptive procedure (Levitt, 1971) to estimate the masker level yielding 50% correct recognition of both target *color* and *number*. The initial level for the masker sentence was set at 30 dBA and increased in level by 5 dB for each correct response until an incorrect response occurred and then decreased in level for each incorrect response until a correct response, and so on. This was repeated until the adaptive track had three reversals in direction, at which point the step size was reduced to 1 dB and six more reversals were obtained. The threshold masker level for a given block of trials was estimated as the average of the last six reversals, and the TMR was calculated by subtracting the masker level from the target level of 60 dBA. The threshold for each condition was computed as the average across the two separate runs. All statistical analyses were conducted in SPSS (version 25, IBM, Armonk, NY).

## 3. Results

Figure 1(A) shows mean TMR values (±1 standard deviation) on the ordinate as a function of the spatial separation on the abscissa with the four target-masker gender combinations indicated by separate symbols. Note that smaller, or more negative, TMR thresholds indicate better (or improved) speech recognition ability. For the co-located (target at 0°, maskers at 0°) spatial condition (leftmost symbols), the same-gender target-masker conditions, MM (shaded square, 2.6 ± 0.8 dB) and FF (shaded circle, 2.6 ± 0.8 dB), exhibit larger TMR thresholds than the different-gender conditions, MF (open triangle, −7.0 ± 3.0 dB) and FM (open diamond, −8.5 ± 2.1 dB). Lower TMR values for different-gender conditions (open symbols) relative to same-gender conditions (shaded symbols) are indicative of VGRM. Relative to the collocated 0° condition, spatial separation of the maskers from ±15° to ±60° (left to right) relative to the target at 0° led to smaller TMR thresholds for all target-masker gender combinations. This reduction is indicative of SRM. The TMR thresholds at the target and masker separation of ±60° (rightmost symbols) were −10.2 ± 2.3 dB for MM, −13.9 ± 2.5 dB for MF, −13.7 ± 2.6 dB for FF, and −15.9 ± 1.7 dB for FM.

A two-way repeated-measures analysis of variance (RMANOVA) was performed with TMR thresholds as the dependent variable and spatial separation (0°, ±15°, ±30°, ±45°, and ±60°) and voice-gender difference (FF, FM, MM, and MF) between target and makers as independent variables. The results showed significant effects of both spatial separation (F_4,76_ = 416.4, *p* < 0.001, η^2^ = 0.956) and voice-gender difference (F_3,57_ = 289.1, *p* < 0.001, η^2^ = 0.938) as well as a significant interaction between these two factors (F_12,228_ = 30.4, *p* < 0.001, η^2^ = 0.615).

The primary goal of this study was to investigate the TMR interaction between voice-gender difference (VGRM) and spatial separation (SRM). To do so, first the VGRM was computed as the TMR difference between the same-gender target-masker conditions and different-gender target-masker conditions (i.e., MM – MF and FF – FM) at each spatial separation (0°, ±15°, ±30°, ±45°, ±60°). The red dashed lines and text in Fig. 1(A) illustrate the VGRM for the female target conditions at the target – masker separation of ±60° (i.e., FF_±60_ – FM_±60_ = 2.2 dB). Second, the SRM was computed as the TMR difference between each of the spatially separate conditions and the co-located condition for the same-gender conditions (MM and FF). The blue dashed lines and text in Fig. 1(A) illustrate the SRM for the female talkers and a target and masker separation of ±60° (i.e., FF_0_ – FF_±60_ = 16.3 dB). Finally, the gray dashed lines and text illustrate the combined release from masking (VGRM + SRM) for a female target as the sum of the SRM (FF_0_ – FF_±60_) and the VGRM (FF_±60_ – FM_±60_), which also can be computed as VGRM + SRM (FF_0_ – FM_±60_ = 18.5 dB).

Given these examples, note that gender-related masking release can be computed separately for male talkers using the MM reference condition and for female talkers using the FF reference condition. Each gender-related masking release can be computed for each spatial condition. In combination, these give the gender-related differences in VGRM or SRM as noted below with k indicating target and masker spatial separation (k = 0°, ±15°, ±30°, ±45°, or ±60°). VGRMs account for gender-difference benefits for male talkers [mean(MM_k_ − MF_k_, MM_k_ − FM_k_)] and female talkers [mean(FF_k_ − FM_k_, FF_k_ − MF_k_)]. SRMs account for spatial separation benefits for male talkers (MM_0_ − MM_k_) and female talkers (FF_0_ − FF_k_). Combined release from masking (VGRM + SRM) accounts for benefits from gender difference and spatial separation for male talkers [mean(MM_0_ − MF_k_, MM_0_ − FM_k_)] and female talkers [mean(FF_0_ − FM_k_, FF_0_ − MF_k_)].

Figures 1(B) and 1(C) show the three different masking release metrics VGRM (blue), SRM (red), and VGRM + SRM (black) as a function of spatial separation for the male-talker reference [Fig. 1(B)] and for the female-talker reference [Fig. 1(C)]. For the male-talker reference condition [Fig. 1(B)], the VGRMs *decreased* from 10 to 4.6 dB as spatial separation increased from 0° to ±60°. Conversely, the SRM *increased* from 0 to 12.9 dB as spatial separation increased from 0° to ±60°. These gradual shifts in the VGRM and the SRM resulted in an intersection of the red and blue functions between ±15° and ±30° spatial separations. The masking release based on combined voice-gender difference and spatial separation (VGRM + SRM) increased from 10.3 to 17.5 dB as spatial separations increased. Similar changes in masking release with spatial separation were observed in the female-talker reference condition (VGRM: 10 to 1.4 dB; SRM: 0 to 16.3 dB; VGRM + SRM: 10.3 to 17.5 dB as spatial separations increased from 0° to ±60°), but the VGRM and SRM intersected at a smaller spatial separation, at around ±15°, than for the male-talker reference condition.

A three-way RMANOVA was computed with magnitudes of masking release as the dependent variable and masking release type (VGRM, SRM, or VGRM + SRM), spatial separation (0°, ±15°, ±30°, ±45°, and ±60°), and talker-gender reference (male and female) as independent variables. The results showed significant main effects of masking release type (F_2,38_ = 460.1, *p* < 0.001, η^2^ = 0.960) and spatial separation (F_4,76_ = 107.9, *p* < 0.001, η^2^ = 0.850) and significant interactions among three independent variables (*p* < 0.001, η^2^ > 0.321 for all cases) except for the interaction between spatial separation and talker-gender reference (*p* = 0.879, η^2^ = 0.015). *Post hoc* pairwise comparisons using Bonferroni correction were computed to better understand the interaction among these three independent variables. The results demonstrated that the magnitudes of VGRM, SRM, and VGRM + SRM were significantly changed as spatial separation increased from 0° to ±60° (*p* < 0.001 for all cases). The results also demonstrated that VGRMs and SRMs were significantly different between male-talker and female-talker references (*p* < 0.001 for both cases), but no significant difference was observed for the VGRM + SRM condition (*p* = 0.781).

## 4. Discussion and conclusion

The TMR thresholds shown in Fig. 1(A) indicate substantial changes in speech reception threshold across the different spatial separations and target-masker gender difference conditions tested. In the same-gender target-masker conditions (MM and FF), there was a decrease in the TMR thresholds (from 2.5 to −14 dB) with increasing spatial separation. The same degree of decrease was not observed, however, in the different-gender target-masker conditions (MF and FM). Instead, in these conditions, the TMR thresholds were considerably lower and changed within the range of about −7 to −15 dB) as spatial separation increased. The presence of this smaller decrease in TMR thresholds in the different-gender target-masker conditions is important as it supports the idea that there is an interaction present between voice-gender difference and spatial separation cues in achieving masking release in multi-talker listening environments.

The data from this study are consistent with the overall trends in masking release reported by Gallun and Diedesch (2013), who investigated gender-specific spatial separation masking release with a smaller set of conditions. Their data also revealed greater decrease in TMR thresholds across spatial separations in the same-gender conditions (7 dB) in comparison to the different-gender conditions (2 dB). By comparison, the TMRs in the current data set (for all of the target-masker gender conditions) were lower than those reported by Gallun and Diedesch (2013), likely due to the younger age and smaller age range of the current subjects (mean 22 ± 1.8 years) relative to those of Gallun and Diedesch (mean 50 ± 15.6 years). It should be noted that Gallun and Diedesch (2013) focused only on variation in TMR thresholds by target-masker spatial separation and did not consider that by target-masker gender difference.

The main goal of this experiment was to investigate the contribution of voice-gender difference cues relative to spatial separation cues to masking release. The current data illustrate parameter-specific changes in the weighting of voice and spatial cues as a function of spatial separation and a single point of intersection where the magnitude of masking release was the same for the two cue types. In Figs. 1(B) and 1(C), the clear intersection of VGRM and SRM occurs at a separation slightly less than ±30° for the male-talker condition and slightly less than ±15° for the female-talker condition. At these crossover points, weighting of VGRM and SRM leads to comparable masking release. For spatial separations less than the intersection points, voice-gender difference cues are the more beneficial cues in the segregation of targets from maskers. Thus, for small spatial differences, VGRM has greater perceptual weighting than SRM. Conversely, at spatial separations larger than those intersection points, listeners weight the spatial cues greater than the voice-gender difference cues.

The voice gender-related differences in masking release observed here deserve further consideration. Both the F0 and VTL are primarily associated with the perceptual characteristics of voice gender by NH listeners (Darwin *et al.*, 2003; Mackersie *et al.*, 2011). With respect to the CRM stimuli used in this study, we used the average power spectral density for all CRM speech stimuli to investigate acoustic correlates of F0 and VTL. The F0 was estimated by the first peak of the spectral envelope (Flanagan, 1965), and the spectral slope was estimated by the slope of the regression line fitted to the spectral envelope using the least square error method (Simantiraki *et al.*, 2018). The result showed clear F0 differences between male (F0: 100 ± 7 Hz) and female (F0: 204 ± 12 Hz) talkers but similar spectral slopes among talkers (male talkers: −14.3 ± 0.3 dB/octave; female talkers: −14.1 ± 0.1 dB/octave). The masking release benefits from the female talkers show faster transition between VGRM and SRM relative to the male talkers, although the overall amounts of masking release (VGRM + SRM) are relatively similar between male and female talker conditions. Darwin *et al.* (2003) also reported that speech segregation performance was higher in conditions when the target talker had either a higher F0 or a smaller VTL, representing a more female-sounding voice. From this, the authors suggested that it may be easier to attend to female than male target speech messages, consistent with the data reported here. Note that the results in Fig. 1(A) show that lower TMR thresholds were observed in female target conditions (FF and FM) compared to male target conditions (MM and MF) across all spatial separation configurations tested in this study.

In summary, the results of this study demonstrate the interactions between voice-gender difference cues and spatial separation cues in multi-talker listening environments and clearly indicate that there is an unequal perceptual weighting between the cues that lead to VGRM and those that lead to SRM. In both males and females, when VGRM and SRM are mapped across a spatial field, there is a point within that field where the benefit is equally elicited by VGRM and SRM. For females, intersection at which the dominant perceptual benefit switches from VGRM to SRM can be elicited at smaller spatial separations than with the male talker. This result indicates that when considering SRM alone, greater spatial masking release will be obtained with a female-talker reference than with a male-talker reference. It is well known that deficits of speech-in-noise performance in hearing-impaired (HI) listeners are likely a consequence of many factors such as reduced audibility, degraded localization ability, and poor spectral and temporal resolution. Future work with HI listeners could provide a more complete understanding of individual differences in the perceptual weighting between voice-gender difference and spatial separation cues and how that weighting might be impacted by hearing loss. In addition, future studies utilizing CRM stimuli that can yield both VGRM and SRM may be more reflective of natural conversation, can maximize the opportunity for auditory stream segregation, and may represent a set of conditions that is more sensitive to speech communication deficits than VGRM or SRM alone.
